# Supplementary material for: Trends in degenerative lumbar spinal surgery during the early COVID-19 pandemic in Republic of Korea: A national study utilizing the national health insurance database
Source: PLoS One. 2024 Jun 11;19(6):e0305128. doi: 10.1371/journal.pone.0305128 (PMC11166321; doi:10.1371/journal.pone.0305128)
Supplement: Attachment — Submitted filename: Response to reviewers.docx [file pone.0305128.s001.docx]

**Response to reviewers**

We deeply appreciate the opportunity to revise our manuscript following the thoughtful and constructive comments from the reviewers. Their feedback has been invaluable in enhancing the quality, clarity, and impact of our work. We hope that the revised manuscript will be deemed suitable for publication, offering valuable contributions to the field and the journal's readership. We look forward to the possibility of our work being published in PLOS ONE and are available for any further information or clarifications as needed.

**Reviewer #1**

**Comment -** Thank you for submitting this interesting and informative manuscript to PLOS ONE. I was pleased to receive it as a reviewer.

**Authors’ response:**

Thank you for your time and effort to review our manuscript. We truly appreciate your valuable comments.

**Comment -** While your manuscript provides valuable insights into an important healthcare topic, there are a few areas that could be refined to further augment the quality and impact of the work. Here are some respectful suggestions that could potentially improve the paper if you choose to implement them:

**Authors’ response:**

We are thankful for your insightful comments, which have significantly contributed to the enhancement of our manuscript, particularly in areas concerning healthcare system and infrastructure, policies across different countries, clinical implications, and future healthcare crisis strategy. We wholeheartedly agree with your perspective. It was indeed a great pleasure to revise our manuscript in line with your valuable suggestions, and we hope that our revisions will fulfill your expectations and meet the standards for publication.

**Abstract**
**Comment #1** - The abstract clearly summarizes key aspects, but the conclusion may be strengthened by elaborating on the practice-relevant implications regarding elective surgery policies during pandemics.

**Authors’ response:**

Thank you for your constructive feedback on our abstract. We appreciate your suggestion to elaborate on the practice-relevant implications of elective surgery policies during pandemics. During the pandemic, COVID-19 patients were primarily treated in tertiary hospitals, allowing lumbar surgeries to proceed with minimal disruption in other types of healthcare facilities. This pattern, while not initially a strategic approach, demonstrated an effective allocation of medical resources under the national health insurance system. These findings, emerging from the challenges posed by the pandemic, offer valuable insights for future planning. Employing such a triage approach could serve as a deliberate strategy in managing elective surgeries during future health crises. In our revised conclusion, we have highlighted the observed effect, which acted like a natural triage system.

**Change to Text**:

[Abstract, Page 3, Line 63-69]

Despite the impact of epidemic waves on monthly surgery numbers, a subsequent compensatory increase was observed, indicating that surgical care has adapted to the challenges of the pandemic. This adaptability, along with the stable total number of operations, highlights the potential for healthcare systems to continue elective spine surgery during public health crises with strategic resource allocation and patient triage. Policies should ensure that surgeries for degenerative spinal diseases, particularly those not requiring urgent care but crucial for patient quality of life, are not unnecessarily halted.

**Introduction**

**Comment #2** - The introduction effectively covers the background of the study. As the discussion notes study limitations regarding generalizability, the introduction could benefit from briefly mentioning upfront that the healthcare infrastructure and pandemic responses likely differ across countries.

**Authors’ response**:

Thank you for your comment regarding the need to clarify the context of healthcare infrastructure and pandemic responses across countries. We acknowledge that our findings are specific to the ROK's healthcare system, which is characterized by universal coverage through national health insurance and medical aid. We have included a brief mention of the ROK's healthcare infrastructure, which, while not universally applicable, serves as a conceptual framework for planning in diverse healthcare systems. We also have included the distinct pandemic response of ROK, different from other countries.

**Change to Text**:

[Introduction, Page 4, Line 81-89]

While the main guidelines and strategies in every country were similar, the healthcare infrastructure and pandemic responses, including spinal surgery varied among countries. [12, 13] For example, in ROK, all people are beneficiary of national health insurance or medical aid, and the access to any hospital is not limited to place. During the COVID-19 pandemic, the government of ROK did not restrict elective surgical procedures officially. On the other hand, in the USA, the COVID-19 pandemic led to a nationwide suspension of elective surgery, including spinal procedures, from March 2020 to May 2020.[14] the COVID-19 pandemic led to a nationwide suspension of elective surgery, including spinal procedures, from March 2020 to May 2020.[14]

Although the government of ROK did not officially restrict elective spinal surgeries during the pandemic, the conditions for such surgeries changed significantly.

**Comment #3** - Consider further elaborating on the rationale for focusing specifically on degenerative lumbar spinal diseases and surgeries. For example, you could explain if patients with these conditions could be more vulnerable during pandemics versus other spine issues.

**Authors’ response**:
We are grateful for the opportunity to clearly explain the focus of our study on degenerative lumbar spinal diseases and surgeries. As briefly mentioned in the introduction part of the original manuscript, degenerative lumbar surgeries are performed not for treating an immediate threat to life, but for improving patients’ quality of life, by alleviating pain and functional impairment. However, during a pandemic, when medical priorities shift towards acute critical care for COVID-19 patients, these lumbar elective surgeries are prone to delays or cancellations, consequently rendering them significantly vulnerable. Even compared to other spine issues, such as cervical or thoracic spinal disease, or spinal trauma, the medical priority of degenerative lumbar disease is low. While postponements may seem appropriate considering the short-term urgency of a pandemic, they can cause increased suffering for patients with degenerative spine issues as the pandemic becomes protracted, even if their conditions are not directly life-threatening. We have further elaborated on these aspects in the introduction part as suggested by the reviewer.

**Change to Text**:

[Introduction, Page 5, Line 99-109]

Surgical interventions for degenerative lumbar spinal disease are typically elective and focused on enhancing quality of life rather than addressing emergencies that threaten life immediately. As a result, these conditions are often assigned a lower priority compared to other spinal diseases such as cervical or thoracic lesions with neurological deficits or spinal traumas requiring urgent surgical interventions. In the initial stages of the COVID-19 pandemic, elective surgeries for degenerative lumbar diseases were frequently deferred or canceled as medical priorities shifted to pandemic management. This practice, although intended to conserve medical resources for acute pandemic care, was found to potentially worsen the long-term quality of life of these patients, increasing their vulnerability as the pandemic protracted. [12, 14-19] Based on this experience, this study was designed to observe the impact of the COVID-19 pandemic on surgeries for patients with degenerative lumbar spinal disease who are vulnerable during pandemics.

**Methods**
**Comment #4** - The methods are detailed overall. As a suggestion, it could be useful to state if there were any changes to hospital or surgical protocols between the cohorts that could have impacted the results through unmeasured confounders.

**Authors’ response**:

Thank you for your comment regarding the potential impact of changes in hospital or surgical protocols on our results. We analyzed the NHID customized data, representing a claim dataset of entire national population. Although it lacks detailed clinical data, our objective was to examine the trends of elective lumbar surgery influenced by numerous COVID-19 pandemic-related factors, including any modifications to hospital or surgical protocols. We specifically looked for changes in hospital or surgical protocols unrelated to the COVID-19 pandemic that could serve as significant confounders. However, we couldn’t find any protocol change between the cohorts during the study period that would qualify as unmeasured confounders separate from those associated with the COVID-19 pandemic. We have acknowledged this in the limitations, noting the potential for confounders related to changes in hospital and surgical protocols, although we were not able to identify specific changes. Again, thank you for raising an important question.

**Change to Text**:

[Limitation, Page 25, Line 483-487]

First, the secondary data lacked information on clinical characteristics, and the exact causes of change in surgical procedures, LOH, and increased costs could not be established. The unresolved issue warrants further analysis of clinical data. In particular, changes in hospital and surgical protocols during the pandemic period could act as potential unmeasured confounders, although we were not able to identify specific changes.

**Comment #5** - Consider providing more details on the propensity score matching process. Which specific variables were included and why? How was the greedy matching algorithm optimized?

**Authors’ response**:

Thank you for your question concerning the details of the propensity score matching process. Initially, our model employed propensity score matching to compare the pre- and post-COVID-19 cohorts. However, based on the reviewers' feedback, highlighting concerns about the appropriateness of the propensity score matching, and on suggestion from another reviewer, we decided to change our comparison approach to linear regression in the whole population with multivariable adjustment. This revised method could be more suitable for comparison between the pre-COVID-19 and COVID-19 periods while enabling us to effectively control for potential confounders. We believe that this modification better aligns with the study's objectives.

**Change to Text**:

[Materials and methods, Page 13, Line 246-250]

A logistic regression model was used to compare the rates of reoperation and readmission within 30 days after surgery between pre-COVID-19 and COVID-19 periods. For multivariate analysis, adjustments were made for age, sex, CCI, income level, length of hospital stay, Parkinson’s disease, osteoporosis, type of insurance, and type of hospital. Crude and adjusted odds ratios (ORs), along with their 95% confidence intervals, were estimated.

**Comment #6** - Consider explaining the rationale for replacing surgery numbers for holiday weeks with average values. Elaborate on assumptions and implications.

**Authors’ response**:

Thank you for your comment. Our rationale for initially replacing surgery numbers during holiday weeks with average values was to minimize the impact of reduced medical service utilization during the Lunar New Year and Thanksgiving. In Korea, these holidays are traditionally accompanied by at least four consecutive days off, and it's common for people to avoid scheduling elective surgeries during these times. However, acknowledging your concern about how this approach might affect the results, we revised our methodology. We no longer replace surgery numbers for holiday weeks with average values; instead, we adjust the weekly number of surgeries by working day. This adjustment would provide a more accurate reflection of surgical activity during these periods without altering the data.

**Change to Text**:

[Materials and methods, Page 12, Line 233-240]

Joinpoint regression analysis was applied to examine changes in time trends of surgical cases over the first year of the COVID-19 pandemic period. This statistical method identifies the time points, called "joinpoints", where linear time trend significantly change and calculated the percentage change between trend-change points, along with the average percentage change in the entire period. The number of spinal surgeries was counted on a weekly basis from January to December 2020, and a 3-week moving average was calculated. To account for the decrease in surgical cases due to weekends or public holidays, such as Lunar New Year and Thanksgiving, the weekly number of surgical cases was adjusted based on the number of working days in those weeks.

**Results**
**Comment #7** - When presenting the changes in proportions of surgery types for the diagnoses, consider speculating on potential reasons driving the shifts. This would give readers more context when interpreting the results.

**Authors’ response**:

Thank you for your valuable comment. In the discussion section of our manuscript, we have briefly mentioned the potential reasons for the shifts in surgery types. Overall, the distribution of surgical procedures did not substantially change. The distribution of surgical procedures for HIVD, however, saw an increase in discectomy and endoscopic discectomy, and a decrease in fusion surgery, which may suggest a pandemic-driven preference for less invasive procedures over more complex surgeries, likely due to concerns regarding extended hospital stays and postoperative care.

Conversely, for LSS and spondylolisthesis, the proportion of fusion surgeries increased, which is inconsistent with the presumed preference toward less invasive surgery. A contributing factor may have been that the reduction in physical activity during the pandemic potentially caused exacerbating symptoms of LSS and spondylolisthesis and prompting patients to proceed with more definitive surgical interventions. The overall increase in surgeries for LSS and spondylolisthesis could also be reflective of this exacerbation. Nonetheless, our dataset lacks detailed clinical information to explain the exact causes of these shifts. Hence, while we can offer plausible hypotheses, the precise reasons for the changes in surgical trends remain elusive. We have included this context in the discussion part.

**Change to Text**:

[Discussion, Page 22, Line 409-419]

Overall, the distribution of surgical procedures did not substantially change. The distribution of surgical procedures for HIVD, however, saw an increase in discectomy and endoscopic discectomy, and a decrease in fusion surgery, which may suggest a pandemic-driven preference for less invasive procedures over more complex surgeries, likely due to concerns regarding extended hospital stays and postoperative care.[14, 36-39] Conversely, for LSS and spondylolisthesis, the proportion of fusion surgeries increased, which is inconsistent with the presumed preference toward less invasive surgery. A contributing factor may have been that the reduction in physical activity during the pandemic potentially caused exacerbating symptoms of LSS and spondylolisthesis and prompting patients to proceed with more definitive surgical interventions. The overall increase in surgeries for LSS and spondylolisthesis could also be reflective of this exacerbation. Nonetheless, since our dataset lacks detailed clinical information, the exact cause of the change remains unexplainable.

**Discussion**
**Comment #8** - The discussion provides meaningful interpretation of results and balances the strengths against limitations. To augment the actionability of the conclusions, you could elaborate further on the presumed practice-relevant implications regarding elective surgery decisions during pandemics.

**Authors’ response**:

Thank you for your insightful suggestion. We recognize that the core criteria for surgical indications and elective surgery decision-making remain unchanged, while the allocation of medical resources and patients' attitudes toward elective surgery become critical in a pandemic setting. We advocate for the application of strict surgical criteria to decide the necessity of elective lumbar surgeries, thereby ensuring that only essential surgeries are performed. This approach should take into account the available medical resources to prioritize effectively among all patients, including those with COVID-19.

**Change to Text**:

[Discussion, Page 21, Line 394-405]

The current study showed that even during the COVID-19 pandemic, total number of elective surgeries for degenerative lumbar spinal disease was maintained, despite the impact of epidemic waves. This result implies that with appropriate allocation of medical resources and medical policies, the core criteria for surgical indications and elective surgery decision-making should be unchanged. Some patients may have exhibited spontaneous improvement during the waiting period, but some may have chosen not to undergo surgery despite their disability not improving. Even those who experienced spontaneous improvement might have benefitted from surgery (e.g., less pain, potentially better neurologic recovery, etc.). Consequently, it is possible that certain patients continued to suffer from pain and disability and were unable to return to their normal lives.[14, 33-35] With applying surgical criteria strictly to decide the necessity of elective lumbar surgeries, essential elective surgeries could be performed. This lesson may be useful in planning policies about spine surgery and transitioning to the “new normal” era.

**Comment #9** - Compare and contrast findings with other COVID-19-era studies on spine surgery trends from different countries to put results into perspective. How and why are they similar or different?

**Authors’ response**:

We thank the reviewer for their suggestion to compare our findings with similar studies from different countries. We have discussed results of similar COVID-19 era studies on spine surgery from other countries in discussion part. As we discussed, most studies from other countries reported a similar significant decrease and delay in elective surgeries during the early COVID-19 era. Detailed situation of each country differ according to their pandemic situation and government’s regulation and policy and it’s difficult to directly compare different results of different countries. Our study presented several distinct findings of ROK. Firstly, the number of COVID-19 patients were relatively small in ROK during the first year of the pandemic, compared to other countries, with three distinct epidemic waves, which allowed us to analyze the specific impact of each wave on surgical trends. Secondly, unlike in some countries, the Korean government did not impose regulatory restrictions on elective surgeries during the pandemic. These unique factors contributed to maintain overall surgical volume despite the pandemic in ROK. To response to your comment, we described more detail about studies from other countries and summarize how they similar and different from ROK.

**Change to Text**:

[Discussion, Page 19, Line 350-371]

After the WHO declared COVID-19 a pandemic in March 2020, most elective procedures were postponed worldwide in order to care for the unprecedented influx of critically ill patients.[12, 15] Ridwan et al. reported that, in Europe, only 15% of respondents in the European Association of Neurosurgical Societies (EANS) replied that elective operations continued during April and May 2020.[31] Blondel et al. reported that, in France, operating rooms for degenerative spine surgery operated at 20 to 50% of normal capacity, with the SFCR (The French Society of Spinal Surgery) guidelines during the early 2020.[12] In the US, after the suspension of spinal surgery from March to June 2020, 47% of 133 patients were re-scheduled by June 2020, 16% were re-scheduled by January 2021, and 37% were ultimately not re-scheduled. Norris et al. also showed that overall, one third of patients whose surgery was suspended at the beginning of the mandatory suspension of elective surgery ultimately did not receive surgical treatment.[14] Patients with neurological dysfunction or severe symptoms were re-scheduled once the mandatory suspension of elective operations was lifted,[14, 32] but about 40% of patients without those problems did not undergo elective surgery.

In contrast, the situation in ROK during the first year of the pandemic showed distinct differences. First, the country experienced a relatively low number of COVID-19 cases compared to others, despite three specific epidemic waves. Second, unlike some countries, the Korean government did not implement regulatory restrictions on elective surgeries during the pandemic. Tertiary hospitals were mostly responsible for treating COVID-19 patients, allowing private hospitals to maintain their surgical volume in terms of medical resources. This redistribution may explain the decreased proportion of elective lumbar surgeries performed in tertiary hospitals during the pandemic (Table 1). Nevertheless, the unprecedented pandemic itself discouraged patients from undergoing elective surgery, particularly during the periods of specific epidemic waves.

**Comment #10** - Discuss the degree to which the conclusions can be reasonably extrapolated to healthcare systems with differing infrastructure and pandemic responses compared to the Republic of Korea population.

**Authors’ response**:

The current results were obtained from data of Republic of Korea, where all people are beneficiary of national health insurance or medical aid. The access to any hospital is not limited to place, disease, and type of insurance. Majority of degenerative lumbar spine surgeries are treated in a private hospital (approximately 90%, Table 1). Therefore, securing hospital bed for COVID-19 patients did not markedly reduce the capacity of treating degenerative lumbar spine surgeries. In addition, COVID-19 patients were cared at tertiary referral hospital. Therefore, the proportion of surgeries decreased by about 1% in tertiary hospitals (Table 1). In this regard, a catch-up increase of surgeries was possible with Korean health insurance system without disrupting urgent care for the COVID-19 patients. However, the proportion of patients in each type of hospital may be different with other disease such as cancer or other complex disease. Therefore, the current idea of allowing or modifying the quota of elective surgeries may be customized based on similar analysis with each disease. In addition, the proportion of degenerative lumbar spine surgeries in large hospitals, where most of COVID-19 patients were cared, may various according to countries and health care system. The current study showed the idea of using secondary data to make plan for contingency and it should not be a generalized plan to every disease, country and health care system. Context of different pandemic response by different countries also has been included according to your previous review comment (comment #9, discussion part line 1578-1614), with mentioning that the findings and conclusions of this study should not be over-generalized to all countries.

**Change to Text**:

[Discussion, Page 24, Line 465-479]

Health care system and infrastructure

The current results were obtained from data of ROK, where all people are beneficiary of national health insurance or medical aid. The access to any hospital is not limited to place, disease, and type of insurance. In the ROK, a significant majority of degenerative lumbar spine surgeries are performed in non-tertiary hospitals (approximately 90%, Table 1). Therefore, securing hospital beds for COVID-19—predominantly at tertiary hospitals—did not markedly reduce the capacity for treating patients with degenerative lumbar spine disease. In this regard, a catch-up increase of surgeries was possible with Korean health insurance system without disrupting urgent care for the COVID-19 patients. However, the proportion of patients in each type of hospital may be different with other disease such as cancer or other complex disease. Therefore, the current idea of allowing or modifying the quota of elective surgeries may be customized based on similar analysis with each disease. In addition, the proportion of degenerative lumbar spine surgeries in large hospitals, where most of COVID-19 patients were cared, may be various according to countries and health care system. The current study showed the idea of using secondary data to make plan for contingency and it should not be a generalized plan to every disease, country and health care system.

**Conclusions**
**Comment #11** - The conclusions concisely summarize the main outcomes. To amplify the impact, you may consider incorporating more specific guidance regarding elective spine surgery policies during future public health emergencies based on your findings.

**Authors’ response**: This study is observational study showing temporary delay or cancellation of surgery number is in turn compensated and total number of surgeries is maintained. Even elective surgery is not an urgent, if the pandemic is persisted more than a year, than it couldn’t be delayed permanently. We showed that elective lumbar spinal surgery could be performed during pandemic period with appropriate distribution of medical resources, and thus we consist that elective lumbar surgery which are necessary and acceptable with medical situation should be performed with appropriate triage, in the future pandemic. Surgery should not be postponed just because it is during the pandemic.

**Comment #12** - Overall, these suggestions aim to enhance the manuscript's overall quality for clinicians, researchers, and healthcare policy makers. I believe that implementing the above suggestions would make your important work more useful for informing practice under crisis situations.

**Authors’ response**:

Thank you for constructive comment. We agree with your opinion that aiming clinicians, researchers, and healthcare policy makers would be make our suggestion more useful. We have included this context to the conclusion.

**Change to Text**:

[Conclusion, Page 25, Line 503-515]
Throughout the early stages of the COVID-19 pandemic, our study observed that while elective lumbar spinal surgeries were initially disrupted by pandemic and epidemic waves, they subsequently experienced a compensatory increase, indicating that surgical care adapted to the pandemic's challenges. This adaptability, alongside the stable total number of operations, highlights the potential for healthcare systems to continue essential surgical services during public health crises with strategic resource allocation and patient triage. As we consider the indirect effects of the pandemic on hospital length of stay, discharge dispositions, costs, and readmission rates, these insights underscore the importance of developing robust, flexible healthcare policies for clinicians, researchers, and healthcare policy makers. Such policies should ensure that elective spinal surgeries, especially those not requiring urgent care but crucial for patient quality of life, are not unnecessarily halted. Although circumstances vary from country to country, making it difficult to generalize these findings universally, the valuable lessons learned from the challenges of the COVID-19 pandemic should be used as a guide for future strategies in managing similar health crises.

**Reviewer #2**

**Comment -** Thank you for the opportunity to review the paper by Kim and colleagues regarding a study on the status of spinal surgery during the COVID-19 pandemic in South Korea using real-world data. This is a well-written manuscript and offers valuable societal insights. I read this manuscript with great interest. While I respect the authors' significant effort in extracting detailed information from nationwide population-based data, there are several methodological problems with this study.

**Authors’ response:**

We are deeply grateful for your thoughtful review of our manuscript and for recognizing the efforts made in this study. Your feedback is highly valued, and we appreciate the attention to detail that has been brought to light. We acknowledge the methodological concerns you have raised and are committed to addressing these issues thoroughly to ensure the robustness and validity of our findings. Your insights not only enrich our study but also contribute significantly to the enhancement of our research. Thank you once again for your constructive critique and for the opportunity to refine our work

Major Comments:
**Comment #1**. The utilization of propensity score matching as a method in this study appears inappropriate, given the assumptions associated with this technique. If the intention is to compare pre- and post-population, I would recommend employing regression adjustment incorporating variables pertinent to the outcome of interest. As alternative methods, standardization methods such as Standardized Mortality Ratios (SMR) could be considered.

**Authors’ response**:

Thanks for your comment. We changed our comparison method from propensity score matching to a logistic regression model to compare the rates of reoperation and readmission within 30 days after surgery between pre-COVID-19 and COVID-19 periods. As you recommended, this revised method could be more suitable for comparing two periods while enabling us to effectively control for potential confounding factors. For multivariate analysis, adjustments were made for age, sex, CCI, income level, length of hospital stay, Parkinson’s disease, osteoporosis, type of insurance, and type of hospital. Crude and adjusted odds ratios (ORs), along with their 95% confidence intervals, were estimated.

**Change to Text**:

[Materials and methods, Page 13, Line 246-250]

A logistic regression model was used to compare the rates of reoperation and readmission within 30 days after surgery between pre-COVID-19 and COVID-19 periods. For multivariate analysis, adjustments were made for age, sex, CCI, income level, length of hospital stay, Parkinson’s disease, osteoporosis, type of insurance, and type of hospital. Crude and adjusted odds ratios (ORs), along with their 95% confidence intervals, were estimated.

**Comment #2**. Overall, the paper is redundant in its description. For example, the "patient inclusion" and "variables" sections have room for improvement.

**Authors’ response**:

Thanks for your comment. I agree with you that some parts of the manuscript seem to be redundant. We have revised methods part of “patient inclusion” and “variables” to address this redundancy. We removed number information in patient inclusion part repeated with figure 1 and removed contents of “Types of diseases and surgical procedures” and move it to patient inclusion, with removal of repeated information as well.

**Change to Text**:

[Materials and Methods, Page 7, Line 159-179]

Patient inclusion

Patients who underwent lumbar spinal surgery with the diagnosis of herniated intervertebral disc disease (HIVD), lumbar spinal stenosis (LSS), spondylolisthesis, and spondylolysis from 2019 to 2020 were selected to investigate trends in spinal surgery using the number of surgical operations per week. These diagnoses were based on the International Classification of Diseases 10th Revision (ICD-10) codes: HIVD (M51.x), LSS (M48.05, M48.06, M48.07, M48.08), spondylolisthesis (M43.15, M43.16, M43.17, M43.18, M53.26), and spondylolysis (M43.06, M43.07, M43.08). When patients had multiple spinal diseases, the representative disease code followed the hierarchical coding algorithm proposed by Martin et al. [24]. For example, if patients were diagnosed with both spondylolisthesis and HIVD, they were categorized as having spondylolisthesis. Surgical methods were primarily divided into decompression only or fusion surgery. For patients with HIVD, decompression surgery was further divided into open discectomy and endoscopic discectomy.

To compare the periods before and during the COVID-19 pandemic, the patients were divided based on the date of lumbar spinal surgery. Patients who underwent surgery from January 1, 2019 to December 31, 2019 were defined as the pre-COVID-19 cohort, and patients from January 1, 2020 to December 31, 2020 as the COVID-19 cohort. The following exclusion criteria were then applied; 1) a history of spinal fracture, infection, cancer, or inflammatory arthritis; 2) previous lumbar spinal surgery; 3) missing data for sex and year of birth; 4) severe disability (class 1 or 2, ambulation was dependent on aids or a wheelchair or the patient was bedridden); and 5) under age 20 years at surgery. Finally, 78,402 patients in the pre-COVID-19 cohort and 80,165 patients in the COVID-19 cohort were included (Table 1 and Fig 2).

**Comment #3**. (Page 7, line 157-) Although many populations have been excluded, **these exclusions may be inappropriate when examining trends in the number of surgeries.**

**Authors’ response**:

Thank you for your insightful comment. I understand your concern regarding the extent of exclusions based on our criteria. To examine the trend of entire spine surgeries, a minimal exclusion approach, as you suggested, might seem more appropriate. However, our study's intent was to specifically investigate surgeries for degenerative lumbar spinal disease in patients who are not severely disabled—those for whom surgery is not mandatory and could be subject to postponement or cancellation due to lower priority during resource reallocation in a pandemic. Our objective was to investigate the trends within this particular patient group, who are especially vulnerable to disruptions in their surgical care during a pandemic. While we agree with your suggestion to consider a broader picture of lumbar spinal surgery trends has merit, please understand the purpose of our research and its value.

**Comment #4**. (Page 15, line 253-) In applying joinpoint regression, the authors appear to have corrected the data, but I cannot determine if this is appropriate. I'm afraid this data handling may affect the results.

**Authors’ response**:

Thank you for your comment. We acknowledge your concern that data handling may affect the results. Thank you for your comment. Our rationale for initially replacing surgery numbers during holiday weeks with average values was to minimize the impact of reduced medical service utilization during the Lunar New Year and Thanksgiving. In Korea, these holidays are traditionally accompanied by at least four consecutive days off, and it's common for people to avoid scheduling elective surgeries during these times. However, acknowledging your concern about how this approach might affect the results, we revised our methodology. We no longer replace surgery numbers for holiday weeks with average values; instead, we adjust the weekly number of surgeries by working day, counting regular weekdays as one working day and Saturdays as a half working day. This adjustment would provide a more accurate reflection of surgical activity during these periods without altering the data.

**Change to Text**:

[Materials and methods, Page 12, Line 233-240]

Joinpoint regression analysis was applied to examine changes in time trends of surgical cases over the first year of the COVID-19 pandemic period. This statistical method identifies the time points, called "joinpoints", where linear time trend significantly change and calculated the percentage change between trend-change points, along with the average percentage change in the entire period. The number of spinal surgeries was counted on a weekly basis from January to December 2020, and a 3-week moving average was calculated. To account for the decrease in surgical cases due to weekends or public holidays, such as Lunar New Year and Thanksgiving, the weekly number of surgical cases was adjusted based on the number of working days in those weeks.

Minor comments
**Comment #1**. Many studies have pointed to a decrease in postoperative hospital stay for various surgeries. Consider citing these.

**Authors’ response**:

Thank you for your constructive suggestion. In response, we have reviewed the literature and incorporated relevant studies that report a decrease in postoperative hospital stay across various surgical fields.

**Change to Text**:

[Discussion, Page 22, Line 420-424]

This study showed a reduced LOS during the COVID-19 pandemic. This aligns with findings from multiple studies across different types of surgery which reported a similar reduction in LOH during the pandemic, including adult spinal deformity surgery [40], elective colorectal surgery[41, 42], brain tumor surgery[43], bariatric surgery[44], and partial nephrectomy[45] without compromising surgical outcomes. Several factors may have contributed to this decrease.

**Comment #2**. (Table 1) The column of CCI is broken; please correct it.

**Authors’ response**:

Thanks for pointing out the broken table. We have corrected it not to broken.

**Comment -** Finally, I would like to express my respect for the great effort of the authors in this paper. I hope this paper will be properly revised and published.

**Authors’ response**:

Thank you for the time and effort you have dedicated to reviewing our manuscript. Your comments have been truly invaluable, and we are grateful for your acknowledgment of our work. We hope that the revisions we made will fully address your insightful feedback and meet the standards for publication.
