# Supplementary material for: Trends in degenerative lumbar spinal surgery during the early COVID-19 pandemic in Republic of Korea: A national study utilizing the national health insurance database
Source: PLoS One. 2024 Jun 11;19(6):e0305128. doi: 10.1371/journal.pone.0305128 (PMC11166321; doi:10.1371/journal.pone.0305128)
Supplement: Attachment — Submitted filename: Response to reviewers.docx [file pone.0305128.s002.docx]

**Response to reviewers**

We greatly appreciate the opportunity to revise our manuscript based on the insightful and constructive comments from the reviewers. We are hopeful that the revised manuscript will be considered suitable for publication, thereby making valuable contributions to the field and the journal's readership. We look forward to the potential publication of our work in PLOS ONE and are available to provide any further information or clarifications as needed.

**Editor**

**Additional Editor Comments –** Thank you for your lots of efforts to improve article. Please confirm comments from Reviewer #2, then reply for the 2 questions.

**Authors’ response:**

Thank you for your time and effort in reviewing our manuscript. We are pleased to meet the standards set by the editor and reviewers. The revision process was a true pleasure for all authors, and we hope our study benefits the academic community.

**Reviewer #1**

**Comment -** Thank you for the attention and consideration you have shown to my suggested revisions for your manuscript. It is evident that a significant amount of effort and thought has been directed towards the refining of your work, integrating the feedback provided during the peer review process. The resulting modifications demonstrate a thorough approach and significantly improve the rigor and overall quality of your manuscript. I look forward to witnessing the impact your research will undoubtedly have on the academic community.

**Authors’ response:**

We sincerely appreciate your time and effort in reviewing our manuscript. Based on your comments and suggestions, we have significantly enhanced the quality of our manuscript and incorporated essential points. Thank you for your valuable feedbacks.

**Reviewer #2**

**Comment -** I would like to thank the authors for their considerable effort regarding the revision. My previous comments were appropriately addressed. Therefore, my decision is provisional accept.

**Authors’ response:**

We truly appreciate your time and effort in reviewing our manuscript and are pleased to meet the journal's standards. The revision process was a great experience for all authors. Thank you.

**Comment #1 –** It seems that something is wrong in the calculation of the number of cases in cohort A in fig 1. 106217-23661-3094-32-702-321=78407 Please confirm this.

**Authors’ response:**

Thank you for pointing this out. We have confirmed that the first number in the flow chart for the pre-COVID-19 cohort was incorrectly entered as the total number of claims instead of the total number of patients. This has been corrected, and the remaining values have been verified for accuracy. We apologize for any confusion caused.

**Change to Text**:

[Original Fig 2]

[Revised Fig 2]

**Comment #2 –** Still, in my computer environment, the column of CCI in Table 1 seems to be broken; please confirm this.

**Authors’ response –** We have re-checked Table 1 on all authors' PCs but could not identify the issue with the broken view. We suggest the editorial office check if there is any problem with Table 1 before publication. We have attached a PDF version of Table 1 for the reviewer to verify the contents. We apologize for any inconvenience. Thank you.
